# Regulation of *cid* and *lrg* expression by CodY in *Streptococcus mutans*

**DOI:** 10.1002/mbo3.1040

**Published:** 2020-04-13

**Authors:** Sang‐Joon Ahn, Hey‐Min Kim, Shailja Desai, Kamal Deep, Kelly C. Rice

**Affiliations:** ^1^ Department of Oral Biology College of Dentistry University of Florida Gainesville FL USA; ^2^ Department of Microbiology and Cell Science Institute of Food and Agricultural Sciences University of Florida Gainesville FL USA

**Keywords:** CcpA, Cid and Lrg, CodY, pyruvate, *Streptococcus mutans*

## Abstract

The ability of *Streptococcus mutans* to persist in a variety of adverse environments and to emerge as a numerically dominant member of stable oral biofilm communities are essential elements for its cariogenicity. The *S. mutans* Cid/Lrg system has been studied as a key player in the integration of complex environmental signals into regulatory networks that modulate virulence and cell homeostasis. Cid/Lrg has also been shown to be closely associated with metabolic pathways of this organism, due to distinct patterns of *cid* and *lrg* expression in response to growth phase and glucose/oxygen levels. In this study, a comparison of *cid* and *lrg* promoter regions with conserved CodY (a regulator which responds to starvation stress)‐binding motifs revealed the presence of a potential CodY‐binding site, which is arranged similarly in both *cid* and *lrg* promoters. Electrophoretic mobility shift assays (EMSAs) and promoter reporter assays demonstrated that expression of the *cid* and *lrg* operons is directly mediated by the global transcriptional regulator CodY. DNase I footprinting analyses confirmed the predicted binding sequences for CodY in both the *cid* and the *lrg* promoter regions. Overexpression of CodY had no obvious effect on *lrgAB* expression, but deficiency of CodY still affected *lrgAB* expression in a *lytST*‐overexpressing strain, suggesting that CodY is required for the full regulation of *lrgAB* by LytST. We also demonstrated that both CodY and CcpA are involved in regulating pyruvate flux and utilization. Collectively, these data show that CodY directly regulates *cid* and *lrg* expression, and together with CcpA (previously shown to directly regulate *cid* and *lrg* promoters) contributes to coordinating pyruvate uptake and utilization in response to both the external environment and the cellular metabolic status.

## Graphical abstract

This article demonstrates the involvement of CodY as a direct regulator of *cid* and *lrg* operons, key players in the integration of complex environmental signals into regulatory networks that modulate virulence and cell homeostasis in *Streptococcus mutans*. It also improves our understanding of the functional links between stress response and cell death/lysis process.

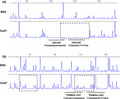

## 1 INTRODUCTION

*Streptococcus mutans* is highly associated with the development of caries, a classic biofilm disease on the tooth surface (Loesche, 1986). The capacity to form biofilms is a major virulence trait of this organism and is closely related to its ability to persist in the oral cavity which experiences dynamic environmental changes such as nutrient limitation (Bowen, Burne, Wu, & Koo, 2018; Burne, 2018; Burne et al., 2012; Koo, Allan, Howlin, Stoodley, & Hall‐Stoodley, 2017; Lemos & Burne, 2008). Regulated cell death and lysis of a bacterial subpopulation which has been damaged by adverse environments have been suggested to be a survival strategy required for the formation of mature pathogenic biofilms (Bayles, 2003, 2007, 2014; Lewis, 2000; Rice & Bayles, 2008). In this respect, we have studied the *S. mutans* Cid/Lrg system, consisting of the two paralogously related dicistronic operons, *lrgAB,* and *cidAB*, encoding predicted membrane‐associated proteins (Ahn, Rice, Oleas, Bayles, & Burne, 2010). The CidA and LrgA proteins share predicted structural features with the bacteriophage‐encoded holin family of proteins, known to be involved in regulating the timing of host cell lysis during bacteriophage lytic infection (Bayles, 2003, 2007, 2014; Groicher, Firek, Fujimoto, & Bayles, 2000; Rice et al., 2003). Interestingly, mutation of the *cid* and *lrg* operons has demonstrated their impact on comprehensive *S. mutans* virulence traits, such as autolysis, biofilm development, oxidative and heat stress responses, antibiotic resistance, and genetic competence (Ahn, Gu, Koh, & Rice, 2017; Ahn et al., 2010; Rice, Turner, Carney, Gu, & Ahn, 2017)*.* Most recently, it has been revealed that *S. mutans* LrgAB functions as a pyruvate uptake system that appears to function during stationary phase (when the primary carbon source is completely exhausted) (Ahn et al., 2019). Pyruvate is a central carbon metabolite and mediates several key pathways, such as the production of acetyl‐phosphate, organic acids and ATP, and the conversion of NAD/NADH. Given that pyruvate can also function as an effective scavenger of ROS, including hydrogen peroxide (H_2_O_2_) (Ahn et al., 2019), this molecule may serve as a central metabolic signal that coordinates the involvement of Lrg in coping with environmental stresses and modulating homeostasis, possibly initiating a metabolic response to induce cell death and lysis in a subpopulation.

Regulation of *S. mutans cidAB* and *lrgAB* expression is quite interesting, in that RNA levels and promoter activities of *cidAB* and *lrgAB* are counterbalanced throughout growth and in response to the availability of glucose and oxygen (Ahn et al., 2010). For example, in the early‐exponential phase, *cid* expression is dominant and *lrg* is repressed. As cells enter the stationary phase, *lrg* activated through the LytST complex, whereas *cid* expression diminishes (Ahn et al., 2010). Expression of the *cid* and *lrg* operons more sensitively responds to glucose levels. In particular, the *lrg* genes are only induced in cultures containing lower levels of glucose (≤15 mM) but are repressed in cultures containing glucose at concentrations of 20 mM and higher (Ahn et al., 2010). This expression pattern is correlated with the capacity of *S. mutans* to take up extracellular pyruvate into the cell at the stationary phase (Ahn et al., 2019). Oxygen levels also influence the magnitude of pyruvate flux during the growth of this organism (Ahn et al., 2019). Our previous study revealed that the expression of the *cid* and *lrg* operons is directly mediated by the global transcriptional regulator CcpA in response to glucose levels (Kim, Waters, Turner, Rice, & Ahn, 2019). Interestingly, two potential *cre* sites (for CcpA binding) in the *cid* promoter are arranged similarly to those in the *lrg* promoter region (Kim et al., 2019). CcpA also appeared to have the opposite effect on the regulation of *cid* (positive) and *lrg* (negative), suggesting that CcpA binding is an important mediator of *cid* and *lrg* transcriptional responses to glucose levels (Kim et al., 2019)*.* Nevertheless, it does not appear that CcpA effects alone are sufficient to understand and explain the observed patterns of *cid* and *lrg* expression in response to glucose since the repression of *lrgAB* promoter activity was not relieved in the absence of CcpA when cells were cultivated in high‐glucose medium (Kim et al., 2019).

CcpA often works together with CodY (a global transcription regulator that participates in starvation adaptation) to sense changes in nutrient availability and coordinates, directly and indirectly, the expression of hundreds of genes involved in carbon and nitrogen metabolism of gram‐positive bacteria (Fujita, Satomura, Tojo, & Hirooka, 2014; Gorke & Stulke, 2008; Kim & Burne, 2017; Santiago, Marek, Faustoferri, & Quivey, 2013; Shivers, Dineen, & Sonenshein, 2006). CodY controls transcription by binding to a moderately conserved 15‐nucleotide (nt) consensus motif (AATTTTCNGAAAATT) in the vicinity of the promoter region of the target genes (Belitsky, 2011; Kim & Burne, 2017; Lemos, Nascimento, Lin, Abranches, & Burne, 2008), by competing with a positive regulator for binding or by serving as a roadblock to RNA polymerase (Belitsky, 2011). The DNA‐binding affinity of CodY is enhanced by its interaction with the branched‐chain amino acids (BCAAs; isoleucine, leucine, and valine) and GTP that act as effector molecules or signals of the nutritional status of the cell (Brinsmade & Sonenshein, 2011; Guedon, Serror, Ehrlich, Renault, & Delorme, 2001; Handke, Shivers, & Sonenshein, 2008; Ratnayake‐Lecamwasam, Serror, Wong, & Sonenshein, 2001; Shivers & Sonenshein, 2004; Sonenshein, 2005). CodY acts mainly as a repressor, and many of the genes which encode components of metabolic pathways are repressed during growth in the presence of excess nutrients and involved in adaptation to poor growth conditions (Sonenshein, 2007). In our previous microarray data, comparing RNA expression profiles of wild‐type and *lytS*‐deficient strains between early‐ and late‐exponential growth phases in BHI medium, it was shown that *codY* was markedly upregulated at early‐exponential phase by about 4.4‐fold and 2.4‐fold in both wild‐type and *lytS*‐deficient strains, respectively, compared to late‐exponential growth phase in both wild‐type and *lytS*‐deficient strains (Ahn, Qu, Roberts, Burne, & Rice, 2012). Thus, we assumed that this growth‐dependent expression of *codY* may be involved in the regulation of *cid* and *lrg* operons and may be, at least partly, responsible for the observed inversely correlated pattern of *cid* and *lrg* expression in response to the growth phase. In this study, we demonstrate that the *cid* and *lrg* promoter regions are direct targets of *S. mut*ans CodY, using electrophoretic mobility shift assays (EMSAs). The potential binding sequences in each promoter region were also identified by DNase I footprinting analyses. We also show that lack of CodY influences pyruvate flux. The data presented clearly indicate that the balanced regulation between *cid* and *lrg* in response to carbohydrate availability and nutritional status of the organism is coordinated by the activities of CodY, together with CcpA, previously shown to directly regulate *cid* and *lrg.*

## 2 METHODS

### 2.1 Bacterial strains and growth conditions

*Streptococcus mutans* UA159 and its derivatives were cultured in brain heart infusion (BHI) medium (Difco Laboratories, Detroit, MI) or chemically defined medium FMC (Terleckyj & Shockman, 1975) containing 11 mM (low‐glucose) or 45 mM (high‐glucose) at 37°C in a 5% CO_2_ incubator. The medium was supplemented by sodium pyruvate (Fisher Scientific) or β‐fluoropyruvic acid sodium salt monohydrate (3FP, Sigma‐Aldrich), as necessary. Antibiotics were used to supplement growth media in the following concentrations: kanamycin (1 mg/ml), spectinomycin (1 mg/ml), and tetracycline (10 μg/ml). For growth measurements, fresh medium was inoculated with 1:100 dilutions of overnight cultures of *S. mutans*. The optical density at 600 nm (OD_600_) was measured at 37°C at 30‐min intervals using a Bioscreen C growth curve analysis system. At least three independent experiments, each in quadruplicate, were performed. A representative result is presented in each corresponding figure.

### 2.2 Mutant construction

A strain constitutively expressing *codY* was constructed as previously described (Ahn & Rice, 2016). Briefly, the promoter region of *codY* (P*codY*) was replaced by a fragment (ΩKm‐P*ldh*) containing a polar kanamycin resistance gene (ΩKm) and an *ldh* promoter region (P*ldh*). For this, two ~0.5‐kb fragments flanking the −35 and −10 sequences of the *codY* promoter were PCR‐amplified, ligated into the ΩKm‐P*ldh* cassette, and transformed into *S. mutans*. The *lytST*‐overexpressing strain (SAB163) was recently constructed (Ishkov, Ahn, Rice, & Hagen, 2020). To delete the *codY* gene in SAB163, we amplified a region containing the tetracycline gene (replacing *codY*) and its flanking arms (~0.5‐kb) in the Δ*codY* strain by PCR and transformed into SAB163. Transformants were selected on BHI agar containing kanamycin (for a strain constitutively expressing *codY*) and tetracycline (for the deletion of *codY* in SAB163), and double‐crossover recombination into each gene was confirmed by PCR and sequencing to ensure that no mutations were introduced into flanking genes. The *codY*‐overexpressing (SAB399) and *codY*‐deficient strains in the *lytST* overexpression background (SAB392) were then each transformed by the P*lrgA‐gfp* construct for conducting the GFP promoter reporter assays described below.

### 2.3 Microplate promoter reporter assay

For measurement of GFP fluorescence of *S. mutans* strains harboring P*lrgA‐gfp* or P*cidA‐gfp* promoter fusion constructs (Kim et al., 2019), their overnight cultures were diluted 1:50 into 1.5 ml of FMC media and grown to an OD_600_ = 0.5. The cultures were diluted 1:50 into 175 μl FMC in individual wells of a 96‐well plate (black walls with clear bottoms; Corning Incorporated) and incubated at 37°C. The optical density at 600 nm (OD_600_) and green fluorescence (excitation 485/20 nm; emission 520/20 nm) were monitored at 30‐min intervals with a Synergy 2 multimode microplate reader (BioTek) controlled by Gen5 software. Fluorescence intensity was calculated by subtracting the fluorescence of wild‐type harboring plasmid without the reporter gene fusion from fluorescence readings of the *S. mutans* strains harboring the P*lrgA‐gfp* or P*cidA‐gfp* gene fusion.

### 2.4 Electrophoretic mobility shift assays (EMSAs) and DNase I footprinting assays

For EMSAs, DNA probes containing the promoter regions of *cid* or *lrg* were amplified by primers labeled on their 5′ end with biotinylated nucleotides. To delete each predicted *cre* site(s) or CodY‐box in *cid* or *lrg* promoter region, biotinylated DNA probes were amplified with primers with the desired base deletions in each promoter sequence. Four fmol of biotin‐labeled promoter regions of *cid* and *lrg* was incubated in combination with the increasing amount (0 and 25 pmol) of purified, recombinant His_6_‐tagged CodY protein (Kim & Burne, 2017) in a 10‐μl reaction mixture containing 10 mM HEPES (pH 7.8), 50 mM KCl, 5 mM MgCl_2_, 1 mM dithiothreitol (DTT), 1 mM EDTA, 1 μg poly (dI‐dC), 1 μg bovine serum albumin (BSA), and 10% glycerol. The binding reaction, sample separation in a polyacrylamide gel, and DNA signal detection were performed as described previously (Kim et al., 2019). To map CodY‐box site(s) in the promoter regions of *lrg* and *cid*, DNase I footprinting assays were also carried out using a nonradiochemical capillary electrophoresis method (Yindeeyoungyeon & Schell, 2000), as described previously (Kim et al., 2019).

### 2.5 Measurement of extracellular pyruvate

*Streptococcus mutans* strains were grown in a low‐glucose (11 mM) FMC medium. Time‐course measurements of extracellular pyruvate during growth were measured as described previously (Ahn et al., 2019). Briefly, for monitoring growth, samples (200 μl) were taken at 1‐ to 2‐hr intervals and half of this volume (100 μl) was used to measure the optical density at 600 nm in a spectrophotometer. The other half (100 μl) was used to quantify extracellular pyruvate concentrations in the supernatant with an EnzyChrom™ Pyruvate Assay Kit (BioAssay Systems). The results are an average of two independent replicates, each performed in duplicate.

### 2.6 Quantitative real‐time PCR (qPCR) assay

To measure the expression of genes using qPCR, *S. mutans* UA159 and Δ*codY* mutant strains were grown in BHI broth at 37°C in a 5% (vol/vol) CO_2_ atmosphere. To measure the growth‐dependent expression of *cidA* and *lrgA* genes, cells were harvested in early‐exponential (EE) and early‐stationary (ES) growth phases. Extraction of RNA, qPCR, and data analysis was performed as described elsewhere (Ahn et al., 2012; Ahn & Rice, 2016; Rice et al., 2017). Expression was normalized against an internal standard (*gyrA*), and data were presented as the relative copy number of mRNA (copies/μl). Statistical analyses were performed on data generated from *n* = 3 independent experiments using an unpaired *t* test.

## 3 RESULTS

### 3.1 CodY is involved in the regulation of *cid* and *lrg* expression

**FIGURE 1:**
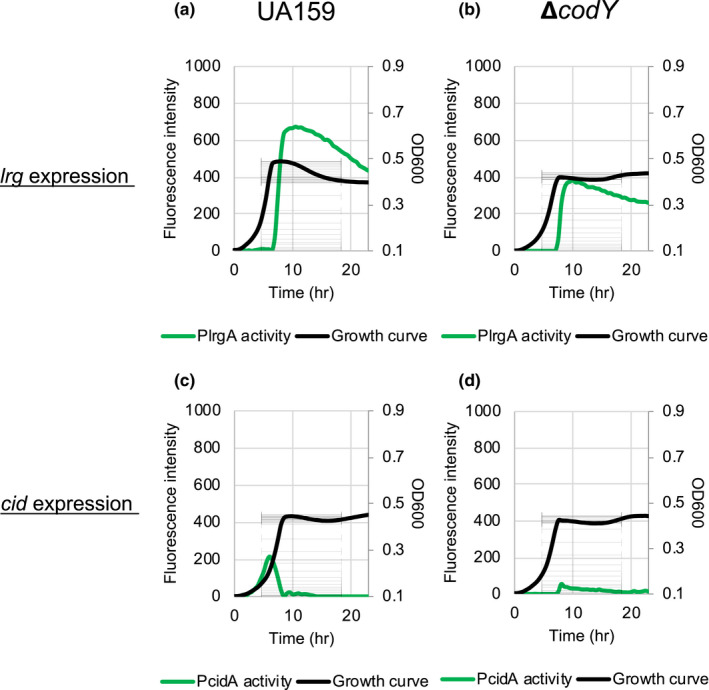
Effect of CodY on *Streptococcus mutans lrg* and *cid* promoter activity over the growth in the low‐glucose cultures. Strains harboring P*lrgA‐gfp* (a and b) and P*cidA‐gfp* (c and d) constructs in pDL278 in the *S. mutans* UA159 wild‐type (a and c) and ∆*codY* (b and d) backgrounds were grown in a chemically defined FMC medium, supplemented by 11 mM glucose. Relative *gfp* expression (colored lines) and OD_600_ (black lines) were monitored on a plate reader (see Materials and Methods for details). The results are representative of three independent experiments

**FIGURE A1:**
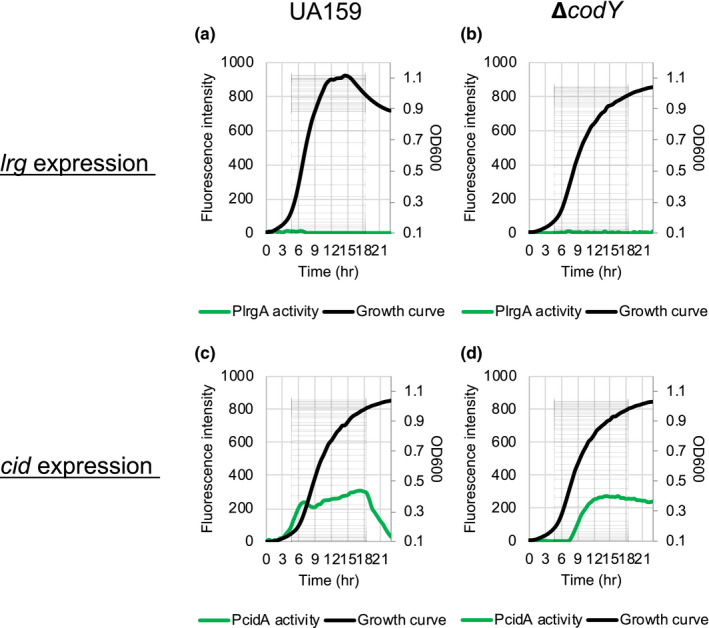
Effect of CodY on *Streptococcus mutans lrg* and *cid* promoter activity during growth in high‐glucose cultures. The strains harboring P*lrgA‐gfp* (a and b) and P*cidA‐gfp* (c and d) constructs in pDL278 in the *S. mutans* UA159 wild‐type (a and c) and ∆*codY* (b and d) backgrounds were grown in a chemically defined FMC medium, supplemented by 45 mM glucose. Relative *gfp* expression (colored lines) and OD_600_ (black lines) were monitored on a plate reader (see Materials and Methods for details). The results are representative of three independent experiments

Given that *lrgAB* expression is induced in stationary phase and its expression is strongly repressed in the presence of excess glucose (Ahn et al., 2010, 2019), we assumed that the global stationary phase regulator CodY (Sonenshein, 2005, 2007) may be involved in the regulation of *lrgAB* and possibly *cidAB*. To assess whether CodY is involved in the regulation of *lrgAB* and *cidAB,* we transformed the P*lrgA‐gfp* and P*cidA‐gfp* reporter constructs, used in our previous study (Kim et al., 2019), into the Δ*codY* mutant strain (Lemos et al., 2008), and monitored the expression of both *lrgAB* and *cidAB* in low‐ and high‐glucose cultures, allowing a strong stationary phase induction of *lrgAB* and *cidAB*, respectively (Ahn et al., 2010; Kim et al., 2019). We first confirmed that *lrgAB* promoter activity is sharply induced at the onset of the stationary phase in low‐glucose cultures (Figure 1a), similar to that reported in our previous studies with this transcriptional GFP reporter construct (Kim et al., 2019). Notably, the absence of the *codY* gene resulted in about a 40% reduction of *lrgAB* expression at the stationary phase (Figure 1b), compared to that of the wild‐type strain (Figure 1a). In high‐glucose cultures, no obvious induction of *lrgAB* was observed in both the absence and presence of CodY (Figure A1a,b in Appendix). In contrast, *cid* promoter (P*cid*) activity was dramatically repressed at stationary phase in the low‐glucose culture of the wild‐type strain (Figure 1c), compared to P*lrg* activity in the same condition (Figure 1a), but *cidAB* showed higher promoter activity during exponential growth compared to stationary phase, as previously reported (Ahn et al., 2010). Notably, exponential phase *cid* induction was also markedly reduced in the absence of the *codY* gene in wild‐type low‐glucose cultures (Figure 1d). In high‐glucose cultures, *cidAB* promoter activity was comparable between wild type and the Δ*codY* mutant strain (Figure A1c,d in Appendix 1). However, the absence of CodY resulted in repression of early‐exponential phase induction of *cidAB* during growth in high‐glucose cultures, compared to that in the wild type. These results suggest that CodY positively regulates the activity of both *lrgAB* and *cidAB* in low‐glucose culture conditions. To verify these read‐outs, we also monitored changes in the expression of *lrg* and *cid* during the transition to stationary phase in BHI, using quantitative real‐time PCR (qRT‐PCR), and observed a similar pattern in *lrgAB* and *cidAB* expression (Table A1 in Appendix 1).

### 3.2 Both *lrgAB* and *cidAB* are regulated by direct binding with CodY

**FIGURE 2:**
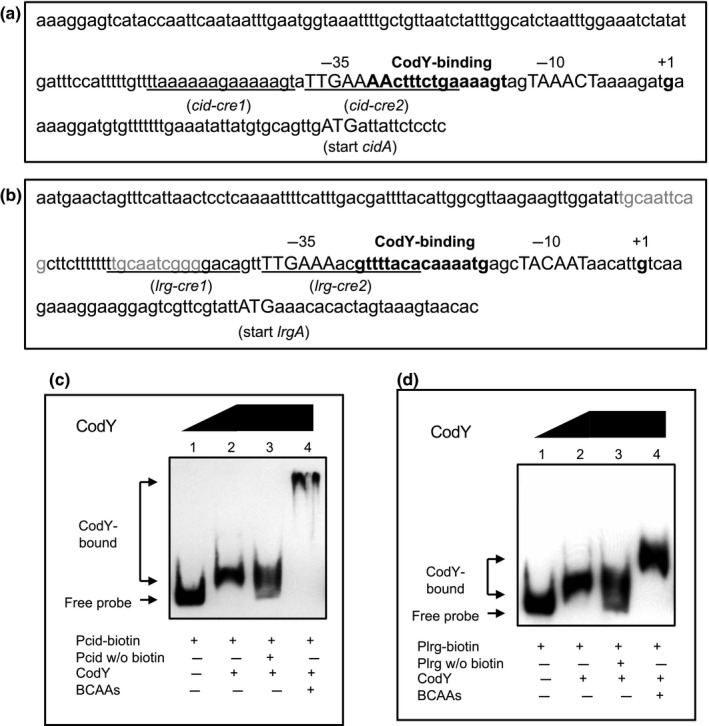
Potential binding site and activity of CodY in the *cid* and *lrg* promoters. a, b; The promoter regions of *cid* (a) and *lrg* (b). The potential CodY‐binding sites are displayed in bold font. The transcriptional start sites (TSSs) are numerically denoted (+1) in bold font, and −10/−35 sequences indicated by capital letters. The hypothetical *cre* sites are underlined. Predicted LytT DNA‐binding motif in the *lrg* promoter region is indicated in gray font. c, d; Electrophoretic mobility shift assays (EMSAs) of CodY binding with the promoter region of *cid* (c) or *lrg *(d). Biotin‐labeled promoter regions (4 fmol) of *cid* (a) and *lrg* (b) were incubated with the increasing amount of purified His‐CodY protein (0 and 25 pmol). Unlabeled promoter regions (0.3 pmol; lane 3) or BCAAs (10 mM; lane 4) were added in the binding reaction mixture, respectively. The reactions were run on a non‐denaturing polyacrylamide gel and the signal observed via chemiluminescence

Given the potential involvement of CodY in the regulation of both *cidAB* and *lrgAB*, we further inspected the promoter region of each operon to identify potential CodY‐binding site(s). In the *cid* promoter region, we discovered a potential CodY‐binding site (AAcTTTCTGAAAAgT) with two mismatches to the consensus sequence, AATTTTCWGAAAATT (Belitsky & Sonenshein, 2008), at positions −30 to −16 with respect to the transcription initiation start (TIS) site of *cidA*, partially overlapped with *cid‐cre2*, recently identified for CcpA binding (Kim et al., 2019), and predicted −35 region of the promoter (Figure 2a). Although we were unable to identify a well‐conserved CodY‐binding site in the *lrg* promoter region, a candidate CodY‐binding site (gtTTTaCAcAAAATg, at positions −30 to −16 with respect to TIS of *lrgA*) was recognized, which contains 5 mismatches to the consensus sequence (Figure 2b). This CodY‐binding site also partially overlaps the *lrg‐cre2* for CcpA binding (Kim et al., 2019) and is located immediately downstream of the predicted −35 region of the promoter (Figure 2b)*.* Next, to determine whether CodY is capable of binding to the *lrg* and/or *cid* promoter regions, including the predicted CodY‐binding site, electrophoretic mobility shift assays (EMSAs) were performed with increasing amounts of purified His‐CodY and biotin‐labeled *cid* or *lrg* promoter regions. The CodY protein bound to both promoter regions of *cid* (Figure 2c, lane 2) and *lrg* (Figure 2d, lane 2), so migration of labeled *cid* and *lrg* promoter fragments was hindered in the presence of CodY. Unlabeled promoter regions of *lrg* and *cid* inhibited the binding of labeled DNA probes to CodY (Figure 2c,d, lane 3, respectively), indicating that the binding interactions are specific. Branched‐chain amino acids (BCAAs; leucine, isoleucine, valine) have been known to be effector molecules for CodY binding to DNA promoter regions in *S. mutans* (Santiago et al., 2013). When BCAAs were used in the binding assays for CodY with the promoter regions of *cid* or *lrg*, 10 mM BCAAs substantially enhanced CodY binding to both *cid* (Figure 2c, lane 4) and *lrg* (Figure 2d, lane 4) promoter regions. These data suggest that interactions between CodY and the *lrg* (or *cid*) promoter occur more effectively in the presence of BCAAs and implicate CodY as another major regulator of *lrg* and *cid* expression, together with CcpA, recently shown to directly bind to the *cid* and *lrg* promoter regions (Kim et al., 2019).

### 3.3 Identification of binding sites for CodY in the *cid* and *lrg* promoter regions

**FIGURE 3:**
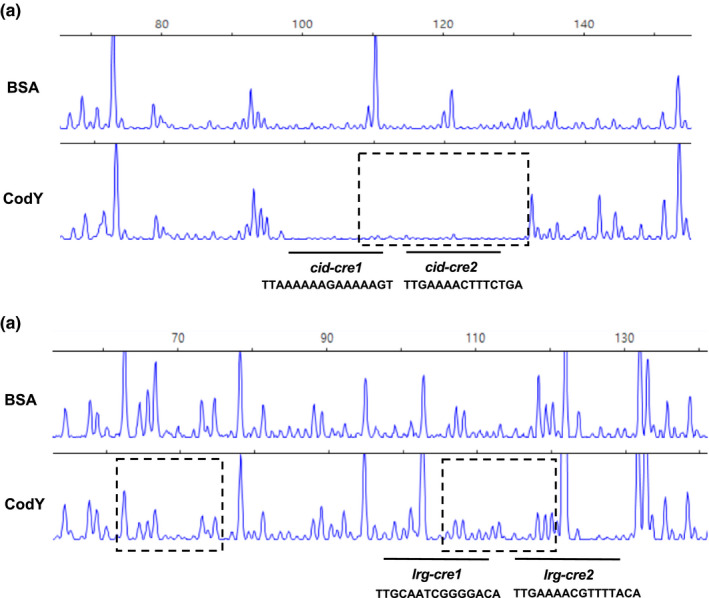
DNase I footprinting assays of CodY binding to the *cid* and *lrg* promoter regions. Dotted boxes depict the regions protected from DNase I digestion upstream of *cid* (a) and *lrg* (b) by CodY. The electropherograms represent control DNA with BSA (bovine serum albumin) on top and footprints with of CodY in the bottom of each panel

**FIGURE 4:**
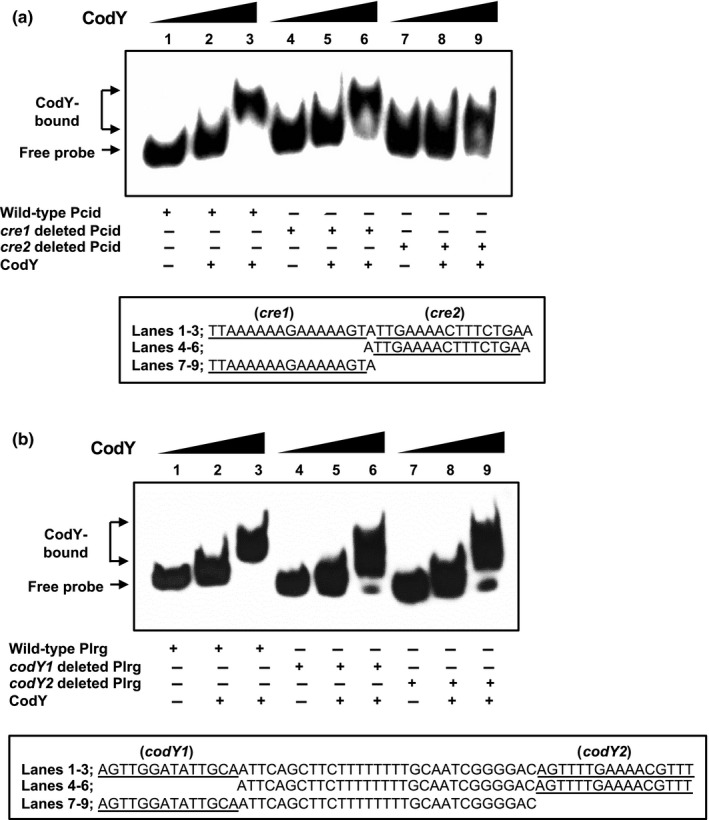
Effects of deleting predicted *cre* and CodY‐box on CodY binding to the *cid* and *lrg* promoter regions. Underlined and letters indicate putative CcpA‐binding (*cre1* and *cre2*) and CodY‐binding (CodY1 and CodY2) sequences in the *cid* (a) and *lrg* (b) promoter regions, respectively. EMSAs were performed with 1 fmol biotinylated *cid* (P*cid*) and *lrg* (P*lrg*) regions with a deletion in the putative *cre* and CodY‐box sequences and various amounts of purified His‐CodY. Each binding reaction mixture contains 0, 6.25, or 12.5 pmol of His‐CodY protein. (a) Lanes 1–3 contain biotinylated P*cid* wild‐type DNA probes; lanes 4–6 contain biotinylated *cre1 *deleted DNA probes; and lanes 7–9 contain biotinylated *cre2 *deleted DNA probes. (b) Lanes 1–3 contain biotinylated P*lrg* wild‐type DNA probes; lanes 4–6 contain biotinylated CodY1 deleted DNA probes; and lanes 7–9 contain biotinylated CodY2 deleted DNA probes. The reactions were run on a non‐denaturing polyacrylamide gel and the signal observed via chemiluminescence. Presented results are representative of two independent experiments

EMSAs showed that CodY could directly bind to the promoter region of *cid* and *lrg* (Figure 2c,d, respectively). First, to identify the precise location of binding by CodY on the *cid* promoter region, DNase I footprinting experiments using capillary electrophoresis (fragment analysis) were conducted using a PCR‐amplified 6‐carboxyfluorescein (6‐FAM)‐labeled DNA probe of the *cid* promoter region (201‐bp) bound to CodY under the same conditions as used for EMSAs. Sites protected from DNase I digestion by DNA binding by CodY were visualized as regions lacking discernable peaks compared to a control reaction mixture containing BSA (bovine serum albumin). Reaction mixtures containing CodY yielded only a single region of protection spanning from −77 to −54, with respect to the ATG start site of the *cidA* gene. This protected region corresponded to the predicted *cid‐cre2* (nt −73/−59) and CodY‐binding sites (nt −68/−54) (Figure 3a), showing that two predicted *cre* sites are involved in binding with CcpA, as well as CodY, and provide further support that both CcpA and CodY proteins may work together to coordinate *cid* expression. To investigate the requirement of the protected regions in DNA binding by CodY, the entire *cid‐cre1* or *cid‐cre2* site was deleted, and the binding affinity of CodY to these deletion constructs was measured by EMSA. The binding of the deleted *cid‐cre1* probe by CodY was weak, showing a slight smear of the probe on the gel (Figure 4a, lane 6). When we tested the ability of CodY to bind the probe in which *cid‐cre2* (including part of predicted CodY‐binding site) was deleted, the probe was less bound by CodY, resulting to more smears on the gel (Figure 4a, lane 9), supporting the contribution of the predicted CodY‐binding sequence (Figure 2a). The data show that the *cid‐cre2* site seems to be more important for CodY binding relative to the *cid‐cre1* site and also suggest that CodY and CcpA may have some competitive interactions on the *cid* promoter. In contrast, in the *lrg* promoter region, CodY binding occurred at two separate sites, as revealed by the DNase I footprinting experiment (Figure 3b). The upstream site, designated *lrg*‐CodY1 (AgTTggatAttgca; mismatches are indicated by lowercase letters), mapped to the positions −114 to −101 with respect to the ATG start site of the *lrgA* gene, but mismatched to the consensus sequence (AATTTTCWGAAAATT) (Belitsky & Sonenshein, 2008). The second site, designated *lrg*‐CodY2 (AGTTTTGAAAACGTTT), extended from −70 to −55 with respect to the ATG start site of the gene included a 15‐bp CodY motif (gtTTTaCAcAAAATg; mismatches are indicated by lowercase letters) with five mismatches to the consensus. Since two potential CodY‐binding sites (*lrg*‐CodY1 and *lrg*‐CodY2) were identified in the *lrg* promoter region (Figure 3b), we tested the ability of CodY to bind the probe in which either *lrg*‐CodY1 or *lrg*‐CodY2 was deleted. As shown in Figure 4b, both deletions impeded the full migration of labeled *lrg* promoter region, showing a strong smear of the probe on the gel, although they could not completely interfere with CodY binding. These data suggest that the regions identified by DNase I footprinting (Figure 3) may contribute to the CodY binding to the *lrg* promoter.

### 3.4 Lack of CodY affects stationary phase pyruvate uptake through LrgAB

**FIGURE 5:**
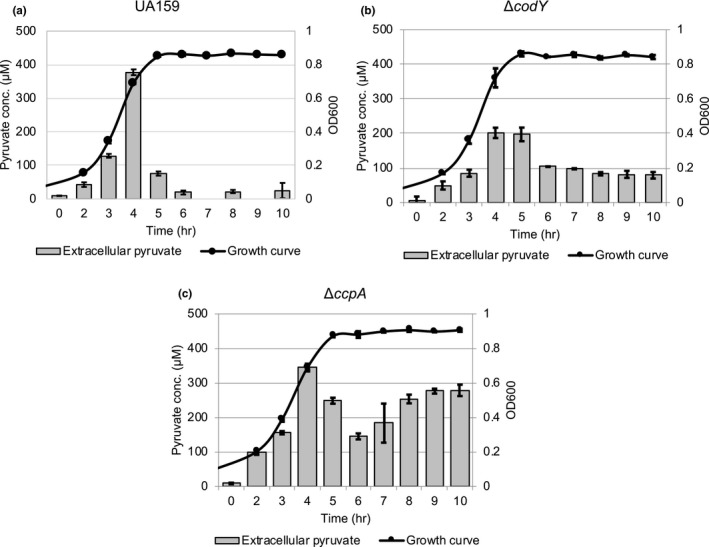
Measurement of extracellular pyruvate during the growth of *Streptococcus mutans* wild‐type,Δ*codY, *Δ*ccpA* strains. The wild‐type UA159 (a), Δ*codY* (b), and Δ*ccpA* (c) strains were grown in the FMC medium supplemented by 11 mM glucose. For time‐course measurements of extracellular pyruvate and growth, samples were taken at 1‐ or 2‐hr intervals (see Materials and Methods for details). The concentration of pyruvate was determined using an EnzyChrom™ Pyruvate Assay Kit and growth was measured by the optical density at 600 nm (OD_600_). Bars indicate the concentration of extracellular pyruvate; and solid line with circles indicates the growth curve. The results are an average of two independent experiments

While the role of CidAB remains enigmatic, we recently reported that LrgAB is responsible for reuptake of pyruvate, excreted during growth as an overflow metabolite (Ahn et al., 2019). Thus, we evaluated whether the transcriptional change of *lrgAB* by lack of CodY affects the capacity of *S. mutans* to take up pyruvate. For this, we monitored the level of extracellular pyruvate during growth in a low‐glucose FMC medium using a Pyruvate Assay Kit (Ahn et al., 2019). In wild‐type culture, pyruvate reuptake initiated at the onset of the stationary phase (Figure 5a), as reported previously (Ahn et al., 2019). In the Δ*codY* strain, however, reuptake of pyruvate was delayed relative to wild type, initiating after about an hour delay and reuptake was overall reduced, with considerable amounts of pyruvate left in the medium (Figure 5b), consistent with the observation that stationary phase *lrgAB* expression is reduced in the *ΔcodY* mutant (Figure 1b). It is also noteworthy that the maximum level (approx. 200 μM) of pyruvate excreted during the growth of the Δ*codY* strain was about 45% reduced compared to wild type, suggesting that lack of CodY may accelerate pyruvate metabolism in the cell, consequently decreasing the amount of overflow pyruvate excretion. Given that the level of accumulated pyruvate is the primary stimulus for *lrgA*B induction and subsequent pyruvate uptake (Ahn et al., 2019), reduced pyruvate excretion (Figure 5b) may be responsible, at least in part, for the reduced stationary phase *lrgAB* expression (Figure 1b) and pyruvate uptake (Figure 5b) in the Δ*codY* strain.

### 3.5 Lack of CodY affects pyruvate flux during exponential growth

**FIGURE 6:**
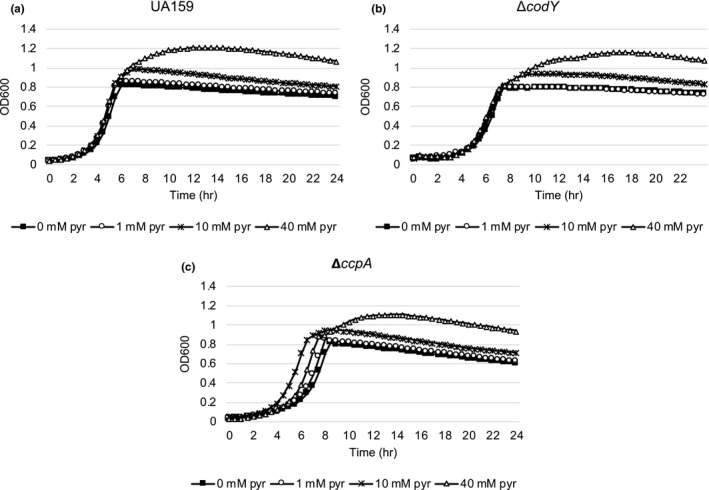
The effect of exogenously added pyruvate on the growth of *Streptococcus mutans* wild‐type,Δ*codY,* and Δ*ccpA* strains. The wild‐type UA159 (a), Δ*codY* (b), and Δ*ccpA* (c) strains were each grown in a low‐glucose (11 mM) FMC medium supplemented by different concentrations of pyruvate (0, 1, 10, and 40 mM). Optical density at 600 nm was monitored every 30 min at 37°C using the Bioscreen C lab system. The results are representative of three independent experiments

**FIGURE 7:**
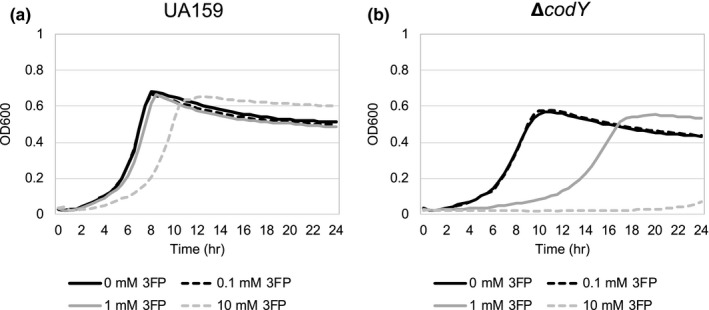
The effect of exogenously added 3‐fluoropyruvate (3FP; pyruvate analog) on the growth of *Streptococcus mutans* wild‐type and Δ*codY* strains. Wild type (a) and Δ*codY* mutant (b) were each grown in a low‐glucose (11 mM) FMC medium supplemented by different concentrations of 3FP (0, 0.1, 1, and 10 mM). Optical density at 600 nm was monitored every 30 min at 37°C using the Bioscreen C lab system. The results are representative of three independent experiments

**FIGURE A2:**
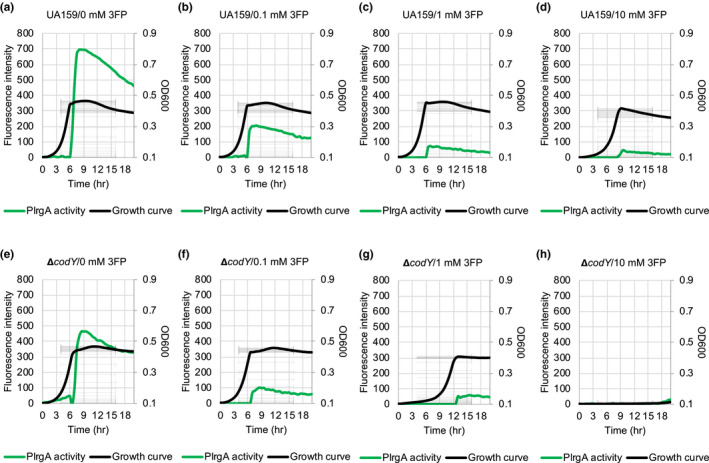
The effect of 3‐fluoropyruvate (3FP; toxic pyruvate analog) on *lrg* promoter (P*lrgA*) activity and cell growth in wild‐type and Δ*codY* strains. The P*lrgA‐gfp* reporter strains in wild‐type (a–d) and Δ*codY* (E‐H) backgrounds were grown in low‐glucose (11 mM) FMC medium supplemented by 0 (a and e), 0.1 (b and f), 1 (c and g), or 10 mM (d and h) 3FP. Relative *gfp* expression (green line) and OD_600_ (black line) were monitored on a plate reader (see Materials and Methods for details). The results are representative of two independent experiments

**FIGURE A3:**
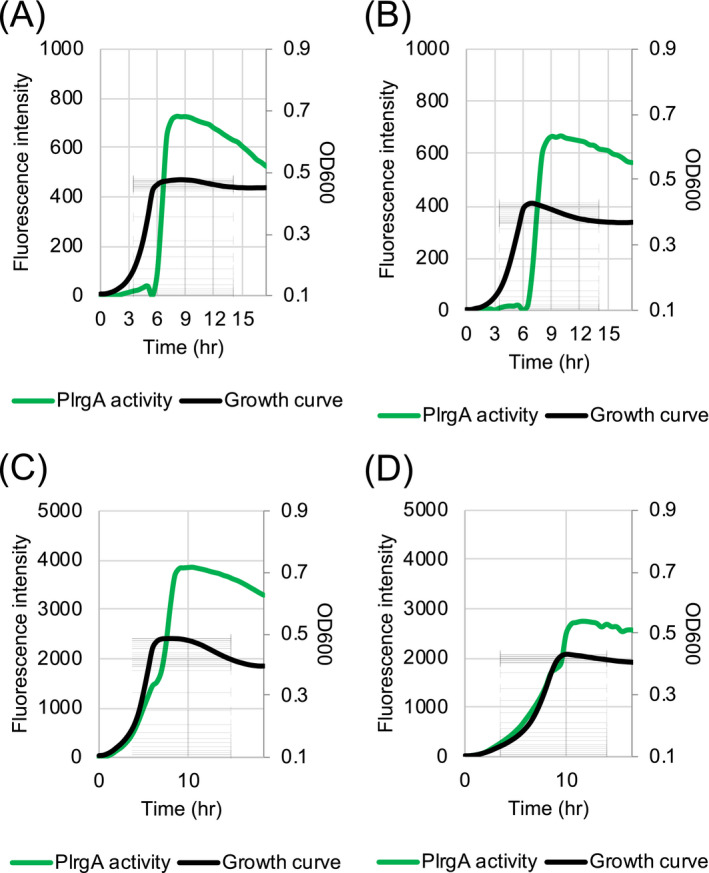
Effect of *codY* or *lytST* overexpression on *Streptococcus mutans lrg* promoter activity during growth in low‐glucose cultures. Strains harboring P*lrgA‐gfp* constructs in pDL278 in the *S. mutans* UA159 wild‐type (a), SAB399 (overexpressing *codY*; b), SAB163 (overexpressing *lytST*; c), and SAB392 (overexpressing *lytST* and lacking *codY*; d) backgrounds were grown in a chemically defined FMC medium, supplemented by 11 mM glucose. Relative *gfp* expression (colored lines) and OD_600_ (black lines) were monitored on a plate reader (see Materials and Methods for details). The results are representative of two independent experiments

We previously showed that supplemented pyruvate had no impact on the growth rate of wild‐type cells but delayed the onset of the stationary phase (Ahn et al., 2019). Therefore, we wondered whether the lack of CodY affects the ability of *S. mutans* to utilize pyruvate during the stationary phase. A similar growth trend to that observed in the wild‐type strain (Figure 6a) was observed in the Δ*codY* strain (Figure 6b), suggesting that the imported pyruvate could be normally utilized for further cell growth when the primary carbon source is exhausted. To further evaluate the effect of *codY* deficiency on pyruvate flux and utilization, we also monitored the growth of wild‐type and *codY* mutant strains in the presence of increasing amounts (0, 0.1, 1, and 10 mM) of 3‐fluoropyruvate (3FP), previously shown to compete with pyruvate and inhibit cell growth by binding to PDH complex in the cell (Apfel, Ikeda, Speckhard, & Frey, 1984; Flournoy & Frey, 1989; Lang, Leystra‐Lantz, & Cook, 1987). As shown in Figure 7a, wild‐type cells displayed normal growth in the presence of 0.1 and 1 mM 3FP, but demonstrated a growth defect in the presence of 10 mM 3FP, as previously observed (Ahn et al., 2019). In contrast, the Δ*codY* mutant was unable to normally grow in the presence of 1 mM 3FP and no growth was observed in the presence of 10 mM 3FP (Figure 7b), suggesting that lack of CodY affected exponential phase pyruvate uptake or utilization, likely in an Lrg‐independent manner. We also cultivated the P*lrgA‐gfp* reporter strain in this same condition to measure *lrgAB* promoter activity. In the wild‐type background, stationary phase expression of *lrgAB* was markedly reduced in the presence of 0.1 mM 3FP (Figure A2a,b in Appendix 1) and almost completely inhibited at concentrations of 1 mM and 10 mM 3FP (Figure A2c,d, respectively, as previously observed (Ahn et al., 2019). Somewhat surprisingly, a similar trend of *lrgAB* expression in response to 3FP was also observed in the Δ*codY* background, although the overall levels of *lrgAB* expression were lower in the Δ*codY* background than in the wild‐type background (Figure A3e–h). Therefore, these results suggest that the growth defect of the Δ*codY* strain by 3FP may be independent of *lrgAB* expression, possibly due to the perturbation of pyruvate transport and metabolism during exponential growth by lack of CodY.

### 3.6 Overexpression of *codY* does not affect *lrgAB* expression

The finding that an identified *lrg*‐CodY1 site (Figure 2b) partially overlaps a predicted LytT‐binding motif in the *lrg* promoter (Kim et al., 2019) suggests a possible interplay between LytT and CodY in activating the *lrgAB* promoter, in agreement with the role of CodY as a positive regulator (Figure 1a,b). To evaluate the role of CodY in regulating *lrgAB*, we created a strain (SAB399), constitutively expressing *codY* from *ldh* promoter in the wild‐type background, as previously described (Ahn & Rice, 2016). Then, we transformed the P*lrgA‐gfp* reporter constructs (Kim et al., 2019) into SAB399 and monitored the expression of *lrg* in the low‐glucose condition. However, overexpression of *codY* had no obvious effect on *lrgAB* expression (Figure A3a,b in Appendix 1), suggesting that the accessibility of LytT to the *lrg* promoter is not fully controlled by CodY. In contrast, overexpression of *lytST* (SAB163) in a wild‐type background led to a dramatic increase of stationary phase *lrgAB* expression (Figure A3c in Appendix 1), as recently reported (Ishkov et al., 2020). To evaluate the effect of CodY on the potential interaction with LytT, we also inactivated *codY* in the *lytST* overexpression background (SAB392), followed by transformation with the P*lrgA‐gfp* reporter construct, and monitored the expression of *lrg* in low‐glucose cultures. As shown in Figure A3d in Appendix 1, deficiency of CodY still reduced the stationary phase expression of *lrgAB* in the *lytST* overexpression strain*,* an expression pattern similar to that observed in the Δ*codY* strain (Figure 1b). These results suggest that the effect of CodY on *lrgAB* promoter activity is independent of LytST.

### 3.7 CcpA is also involved in pyruvate flux and utilization

Since the predicted LytT‐binding motif overlaps predicted CcpA‐binding site (*lrg‐cre1*) in the *lrg* promoter (Figure 2b), we assumed that together with CodY, CcpA may be also involved in the ability of *S. mutans* to take up and utilize extracellular pyruvate. For this, we monitored the level of extracellular pyruvate during the growth of the Δ*ccpA* strain in the same condition used above (Figure 5a,b). As shown in Figure 5c, pyruvate excretion in the *ccpA* mutant was similar to that observed in the wild‐type strain, but reuptake of pyruvate at the stationary phase was marked decelerated in the *ccpA* mutant, with more than 75% of excreted pyruvate left in the medium in stationary phase. This result was quite surprising, because stationary phase *lrgAB* expression was elevated in the Δ*ccpA* strain, relative to the wild‐type strain (Kim et al., 2019). We also evaluated the ability of the Δ*ccpA* strain to utilize pyruvate in the stationary phase by growing *S. mutans* in low‐glucose FMC medium, supplemented by different concentrations of pyruvate, as described above (Figure 6a,b). As shown in Figure 6c, the addition of external pyruvate to the medium effectively prolonged exponential growth of the Δ*ccpA* strain but interestingly, it also enhanced the growth rate of the organism. Taken together, these data suggest that although CcpA regulates stationary phase *lrgAB* expression (Kim et al., 2019), it also appears to affect exponential growth by rerouting pyruvate metabolism.

## 4 DISCUSSION

The ability of *S. mutans* to maintain balanced carbon metabolism in response to environmental conditions, particularly in the transition from conditions that allow exponential growth, to conditions that limit growth, is an important virulence attribute of this organism that promotes its survival and persistence during periods of “feast or famine” in oral cavity. Through our previous studies, we have demonstrated that the Cid/Lrg system is regulated in a growth phase‐dependent manner and by glucose and oxygen levels (Ahn et al., 2010, 2017; Ahn & Rice, 2016). In particular, the findings that the *lrg* genes are specifically induced at stationary phase (Ahn et al., 2010; Kim et al., 2019), and that their gene products are required for reuptake of pyruvate (Ahn et al., 2019), further support the importance of the Cid/Lrg system in preserving cellular homeostasis when exogenous carbohydrate has been depleted in the environment. The findings presented here reveal that the global transcriptional regulator CodY directly regulates transcription of the *cid* and *lrg* genes in *S. mutans*. CodY is known to repress stationary phase gene expression during exponential phase in many bacteria (Sonenshein, 2005). In our previous microarray data for comparison of early‐ versus late‐exponential growth phases in the wild type, it was shown that the *codY* gene was >4‐fold upregulated at early‐exponential phase, compared to that of late‐exponential growth phase (Ahn et al., 2012; Kim et al., 2019), indicating that CodY may actively repress the stationary phase induced genes, including *lrg*, during exponential growth. Given that LytT is required for expression of *lrgAB* and that *lrgAB* expression is reduced in the Δ*codY* mutant strain, we assumed that CodY may be required for full activity of LytT. This idea may be supported by the finding that one of predicted CodY‐binding sites (*lrg*‐CodY1) partially overlaps a predicted LytT‐binding motif in the *lrg* promoter. However, the finding that the overexpression of *codY* had no evident effect on the stationary phase expression of *lrgAB* suggests that the abundance of CodY alone does not affect the accessibility of LytT to the promoter region. Nevertheless, based on the EMSA and DNases I footprinting data, it is likely that CodY may interact with LytT on the *lrg* promoter. Notably, unlike the *codY* gene, the *lytT* gene was >14‐fold downregulated at early‐exponential phase, compared to that of late‐exponential growth phase (Ahn et al., 2012; Kim et al., 2019). Thus, a possible scenario for the interplay between CodY and LytT may be that expression of *lrg* is strongly repressed by CodY during exponential growth, and as the cells approach stationary phase, CodY may interact with LytT in order to activate the *lrgAB* promoter*.* This regulatory switch for *lrg* expression may be affected by intracellular metabolic status. In this study, we showed that BCAA enhanced the binding of CodY to *lrg* (and *cid*) without a further requirement for GTP, suggesting that BCAAs are predominant co‐effectors in mediating an interaction between CodY and *lrg* (or *cid*), and the *cid* and *lrg* operons respond to changes in amino acid availability. Therefore, it is possible that CodY loses its repressing activity as a result of the decrease in the intracellular levels of BCAAs and/or GTP that occurs at the onset of stationary phase, or by interacting with LytT. The levels of BCAAs may reflect the metabolic/nutritional status of cell, allowing CodY to coordinate a variety of genes that are induced when *S. mutans* cells make the transition from rapid exponential growth to stationary phase. However, it does not appear that CodY competes with LytT for the potential binding site on the *lrgAB* promoter, because (a) lack of CodY does not release the repression of *lrgAB* during exponential growth and (b) it still markedly reduces the stationary phase expression of *lrgAB* in the strain overexpressing *lytST*, showing dramatic elevation of *lrgAB* expression at stationary phase. Thus, the access of LytT to the *lrgAB* promoter or its full function as an activator may require CodY at stationary phase. To address this idea, we are currently investigating the potential interaction between CodY and LytT in the regulation of *lrgAB*. Given that CodY is a major repressor of stationary phase gene expression during exponential phase, and deficiency of CodY renders the cell more sensitive to 3FP during growth, it is also possible that CodY may be involved in both carbohydrate and pyruvate metabolism, related to cell growth. In fact, the *S. mutans* Δ*codY* strain grows slowly (Lemos et al., 2008), compared to the wild‐type strain.

More interestingly, the predicted LytT‐binding motif, partially overlapping with the *lrg*‐CodY site, also overlaps the predicted *cre1* site (for CcpA binding) in the *lrg* promoter, indicating that activation of *lrg* by LytT is influenced by both CcpA and CodY. In our previous EMSAs, it is quite apparent that CcpA has a higher binding affinity to the *cid* and particularly *lrg* promoter regions than CodY, unless the level of BCAAs is increased in the culture (Kim et al., 2019). The addition of BCAAs to the EMSA experiments was more effective at promoting CodY binding to the *cid* promoter than to the *lrg* promoter, suggesting that regulation by CodY may overcome regulation by CcpA or RNA polymerase for *cid* expression in the presence of BCAAs. Based on the data so far, it is still premature to speculate on how CodY and CcpA contribute to the expression and function of *cid* and *lrg*, even though it is clear that CodY and CcpA are involved in the regulation of *cid* and *lrg* at a molecular level. Given that CcpA would be activated by an elevated level of glycolytic intermediates (Deutscher, Kuster, Bergstedt, Charrier, & Hillen, 1995; Saier, 1996), competition or cooperation among these regulators may depend on the cellular metabolic status and its surrounding environment. An interesting finding is that supplemented pyruvate can be efficiently utilized even during exponential growth in the Δ*ccpA* strain, although no *lrgAB* induction was observed (Kim et al., 2019), suggesting that CcpA plays an important role in coordinating the utilization of glucose, as well as pyruvate during cell growth. On the contrary, stationary phase pyruvate uptake does not seem to efficiently occur in the absence of CcpA. As well, the overlapped positioning of the *cre* (CcpA binding) and the CodY‐binding sites further support the idea that CcpA and CodY work together in regulating both *cid* and *lrg* expression, and changes in glycolytic metabolite and amino acid pools act as signals that alter the balance between the effects these two regulatory proteins have on *cid* and *lrg* expression. This current study offers an intriguing potential insight toward understanding metabolic linkages with *cid* and *lrg* operons.

In conclusion, our data show that the Cid/Lrg system, presumably integrating signals associated with cellular metabolic status and its surrounding environment, is coordinated through direct interaction with global regulators CodY and CcpA, the latter which has previously been shown to regulate *cid* and *lrg* expression (Kim et al., 2019). Given that lack of CodY affects *lrgAB* expression and excreted pyruvate flux, CodY seems to be closely linked to the uptake and utilization of pyruvate, a key intermediate in various metabolic pathways and rerouted to the synthesis of BCAAs by the action of the *ilv* biosynthetic pathways that are also under the control of CcpA and CodY (Fujita et al., 2014; Santiago, Macgilvray, Faustoferri, & Quivey, 2012; Santiago et al., 2013; Shivers & Sonenshein, 2004, 2005). Given the hypothesized role of Cid and Lrg in inducing cell death and lysis, pyruvate may serve as a metabolic determinant that influences the fate of cells within a population to lyse under unfavorable conditions, which seems to be regulated by LytT and at least two global regulators, CodY and CcpA. From a regulatory standpoint, it is also interesting to determine how *cid* and *lrg* regulation is influenced by competitive interactions among those regulatory proteins. Taken together, this study provides new insights into how the regulation of *cid* and *lrg* expression mediates the response of *S. mutans* to the unpredictable changing oral cavity environment and improves our understanding of the functional links between stress response and cell death/lysis process.

## CONFLICT OF INTEREST

None declared.

## AUTHOR CONTRIBUTIONS

Sang‐Joon Ahn: Conceptualization (lead); formal analysis (supporting); funding acquisition (lead); investigation (supporting); methodology (lead); supervision (lead); validation (supporting); writing‐original draft (lead); writing‐review and editing (lead). Hey‐Min Kim: Formal analysis (lead); investigation (supporting); methodology (supporting); visualization (lead); writing‐original draft (supporting); writing–review and editing (supporting). Shailja Desai: Formal analysis (supporting); investigation (supporting); methodology (supporting); visualization (supporting). Kamal Deep: Formal analysis (supporting); investigation (supporting); methodology (supporting); visualization (supporting); writing–review and editing (supporting). Kelly C Rice: Conceptualization (supporting); funding acquisition (supporting); writing‐original draft (supporting); writing–review and editing (supporting).

## ETHICS STATEMENT

None required.

## Data Availability

All data generated or analyzed during this study are included in this published article.

## Appendix 1

**TABLE A1:** The comparison of pyruvate metabolism‐related gene expression in early‐exponential (EE) versus early‐stationary (ES) growth phases of *Streptococcus mutans* wild‐type and Δ*codY* strains by real‐time qPCR

	Wild type		Fold change (ES/EE)	Δ*codY*		Fold change (ES/EE)
	EE	ES		EE	ES	
*lrgA*	1.28E+03^a^ (9.39E±02)	4.09E+07 (2.34E±07)	32,953	2.27E+03 (1.78E±0.3)	3.06E+06 (2.33E±05)	1,348
*cidA*	1.89E+03 (7.70E±02)	1.55E+02 (8.54E±01)	0.08	1.16E+03 (1.32E±02)	9.41E+01 (6.31E±01)	0.08

^a^ Relative copy number of mRNA (copies/μl); standard deviation in parentheses.
